# Solution Structures of *Bacillus anthracis* Protective Antigen Proteins Using Small Angle Neutron Scattering and Protective Antigen 63 Ion Channel Formation Kinetics

**DOI:** 10.3390/toxins13120888

**Published:** 2021-12-11

**Authors:** Ariel Michelman-Ribeiro, Kenneth A. Rubinson, Vitalii Silin, John J. Kasianowicz

**Affiliations:** 1 Physical Measurement Laboratory, National Institute of Standards and Technology, Gaithersburg, MD 20899, USA; lasirenita54@yahoo.com; 2 NIST Center for Neutron Research, National Institute of Standards and Technology, Gaithersburg, MD 20899, USA; 3 Department of Biochemistry and Molecular Biology, Wright State University, Dayton, OH 45435, USA; 4 SPR Biosystems, Brunswick, MD 21758, USA; vital@sprbiosystems.com; 5 Department of Applied Physics & Applied Math, Columbia University, New York, NY 10027, USA; 6 Freiburg Institute for Advanced Studies, Universität Freiburg, 79104 Freiburg, Germany; 7 Department of Physics, University of South Florida, Tampa, FL 33628, USA

**Keywords:** A-B toxins, alpha-hemolysin, anthrax, *Bacillus anthracis*, bacterial toxin, β-barrel, endotoxin, ion channel, pore forming toxin, PA63, PA83, protective antigen

## Abstract

We are studying the structures of bacterial toxins that form ion channels and enable macromolecule transport across membranes. For example, the crystal structure of the *Staphylococcus aureus* α-hemolysin (α-HL) channel in its *functional* state was confirmed using neutron reflectometry (NR) with the protein reconstituted in membranes tethered to a solid support. This method, which provides sub-nanometer structural information, could also test putative structures of the *Bacillus anthracis* protective antigen 63 (PA63) channel, locate where *B. anthracis* lethal factor and edema factor toxins (LF and EF, respectively) bind to it, and determine how certain small molecules can inhibit the interaction of LF and EF with the channel. We report here the solution structures of channel-forming PA63 and its precursor PA83 (which does not form channels) obtained with small angle neutron scattering. At near neutral pH, PA83 is a monomer and PA63 a heptamer. The latter is compared to two cryo-electron microscopy structures. We also show that although the α-HL and PA63 channels have similar structural features, unlike α-HL, PA63 channel formation in lipid bilayer membranes ceases within minutes of protein addition, which currently precludes the use of NR for elucidating the interactions between PA63, LF, EF, and potential therapeutic agents.

## 1. Introduction

Ion channels and pore-forming toxins have been studied extensively because of their significance in the proper function and disease states of different tissues [1,2,3]. One class of channels is of particular interest because the pore-forming toxins work in concert with other proteins that are produced by their respective bacteria. As an example, *Bacillus anthracis* is unusual in that it secretes two A toxins (lethal factor, LF and edema factor, EF) and one B-toxin (protective antigen 83, PA83) [4,5]. The latter binds to the exterior of cell membranes [6], is cleaved by a furin-like protease to PA63 [7], and one of the products (PA63) forms a heptameric pre-pore complex PA63_7_ [8]. LF or EF associates with PA63_7_, and the A-B complexes of LF-PA63_7_ or EF-PA63_7_ are removed from the cell membrane exterior by endocytotic vesicles, where PA63_7_ channel formation is thought to occur after endosome acidification [9]. PA63_7_ is required for LF and EF to gain entry into the cytoplasm: LF inhibits mitogen-activated protein kinases (which leads to apoptosis), and EF leads to increased cyclic AMP concentration (which leads to cell edema) [10,11]. 

Electrophysiology experiments with PA63 channels in planar bilayer membranes demonstrate that at neutral pH, LF and EF bind reversibly to the cap domain of the channel, with a dissociation constant *K_D_* ≈ 40 pM (where M = mol L^−1^) [12,13,14]. LF or EF bind to the channel to block the ionic current in a diode-like fashion (blocked for positive but not negative applied potentials) [12]. Similar experiments showed that *N*-terminal fragments of LF and EF (LF_N_ and EF_N_, ≈ 30 kg mol^−1^ each) also bind to the PA63_7_ channel [15,16]. When the bulk LF_N_ and or EF_N_ concentration is reduced, under some solution conditions, the channel block by these fragments ceases, which is consistent with a simple reversible binding scheme.

The latter effect has also been interpreted as the unfolding and complete transport of the LF_N_ and EF_N_ fragments through the PA63_7_ channel. By inference, it was suggested that at acidic solution pH (e.g., pH ≈ 5.5), full-length LF (90 kg mol^−1^) and EF (89 kg mol^−1^) would similarly unfold while bound to the channel, completely translocate past the 1.1 nm diameter narrowest constriction in the pore [17,18,19], and refold when they emerge as a whole in the cytoplasm. However, experiments that better mimicked the acidification inside endosomes (i.e., pH 5.5 with LF or EF still in the bulk aqueous phase) suggested that LF and EF become *irreversibly* bound to the PA63_7_ channel cap domain [20], which would inhibit their complete translocation through the pore. This irreversible binding result is consistent with the observation that the complex of LF-PA63_7_ was found in the blood of moribund animals previously subjected to anthrax inhalation [13]. An alternative to the hypothesis of the complete translocation of LF and EF through the PA63 channel was suggested because of the last two experimental results and two other findings: (1) lipid membranes are more likely to rupture at relatively low transmembrane potentials only when either LF or EF became irreversibly bound to the channel [20] and (2) the enzymatic activity of LF alone is only slightly greater than that of the complex of LF-PA63_7_ [13]. The latter further suggests that perhaps it is the *complex* of these two A-B toxins that cause the lethal effects of anthrax infection [13]. However, more research is needed to test these two hypotheses.

Determining the structures of the *B. anthracis* A-B toxins separately, or bound in the complex, should help understand their mechanisms of action and possibly lead to more effective therapeutic agents against them. There are several methods available for this purpose, including x-ray crystallography [21,22,23,24], cryo-electron microscopy (cryo-EM) [25,26,27,28], small angle neutron scattering (SANS) [29], and neutron reflectometry (NR) [30]. 

**Figure 1:**
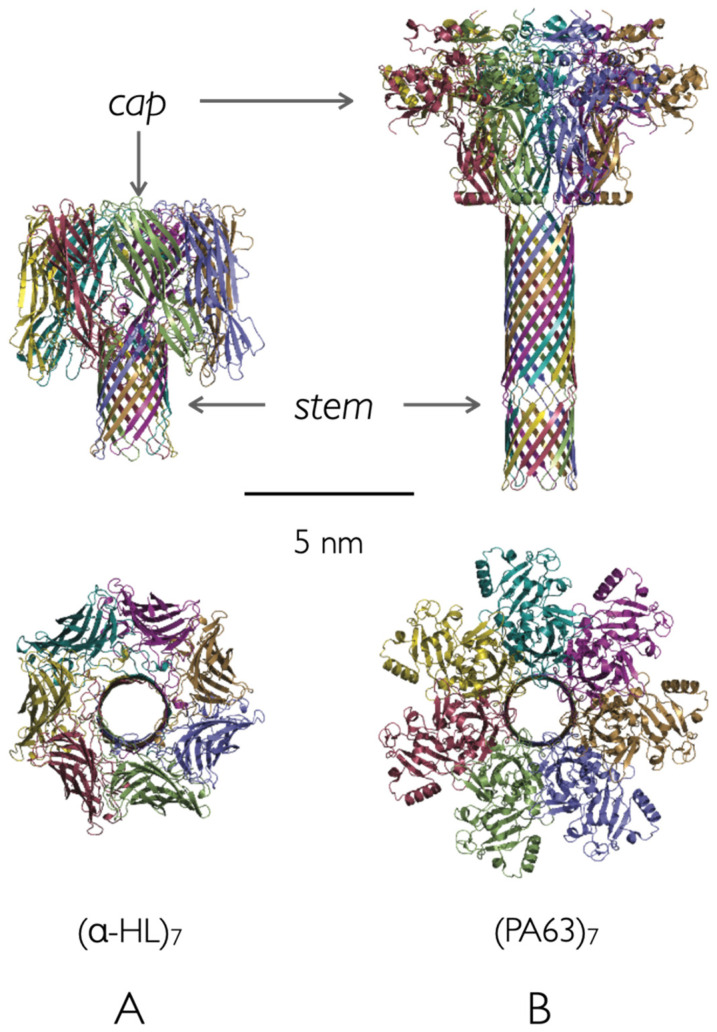
Side and top views of the solution structures of heptameric ion channels formed by the bacterial endotoxins (**A**) *Staphylococcus aureus* alpha-hemolysin and (**B**) activated *Bacillus anthracis* protective antigen (PA63) from PDB 7AHL [23], and PDB 3J9C [48], respectively. The β-barrel segment of the α-HL channel just spans the lipid bilayer membranes. The upper portions of both structures are referred to as the cap domain. The length of the PA63_7_ β-barrel stem section, determined with a combination of site-directed mutagenesis and electrophysiology [49] following the method of Akabas and Karlin [50], is over two-fold greater than the membrane thickness. It was assumed that the segment between the cap domain and the membrane-solution interface interacts with receptors on cell surfaces.

We demonstrated the feasibility of neutron reflectometry for membrane protein structure determination using *Staphylococcus aureus* α-hemolysin (α-HL) [31]. At that time, it was known that α-HL [32] forms heptameric pores [33], and a crystal structure of the heptamer from solution was determined in 1996 [23] (Figure 1A). However, because the α-HL channel has been used in a wide range of single-molecule detection and identification applications [34,35,36,37,38,39,40,41,42,43,44], we wanted to develop a structural method for the molecule in its functional state. To accomplish that, the protein was reconstituted on membranes tethered to a solid support, as had been done by Cornell and colleagues for gramicidin-based sensors [45]. Surface plasmon resonance [46] and electrochemical impedance spectroscopy [31,47] were used to verify that the functional channels had absorbed the tethered membranes. The results demonstrated that the NR structure determination method requires close-packed α-HL channels in the membrane [31]. This was made possible because α-HL spontaneously and continuously forms channels in planar black lipid membranes and tethered membranes without the need for other agents (e.g., detergents).

We report here the use of SANS to determine the structures of *B. anthracis* PA83 and PA63 in solution and electrophysiology to investigate the channel-formation kinetics of PA63 in lipid bilayers. Ultimately, the goal is to test the validity of detailed structural models of the PA63_7_ channel model in solution (with an inferred β-barrel) [48] (Figure 1B) and determine where LF and EF bind to it.

## 2. Results

Neutron scattering can be used to determine the structures of proteins in solution because, as with light, there is contrast between the water molecules and the protein. For light scattering, the contrast is due to a difference in the dielectric constants. With neutrons, the contrast (related to the scattering length density, SLD) is due to the different atom contents in the scatterer (especially the non-exchangeable protons in the protein) and the solvent D_2_O; there is a marked difference in the scattering amplitudes of neutrons by hydrogen and deuterium [51,52,53]. 

In light scattering, the wavelength (≈5000 Å) is much greater than the size of a protein. The interpretation of the results assumes that the protein scatters light as if it were a point source. However, for cold neutrons, such as those used here, their de Broglie wavelength is 5 Å to 6 Å. Because the proteins are larger than this wavelength, the scattering provides information about their shapes. As a result, SANS can be used to study the structures of a wide range of materials, including biological macromolecules [52,54,55]. 

**Figure 2:**
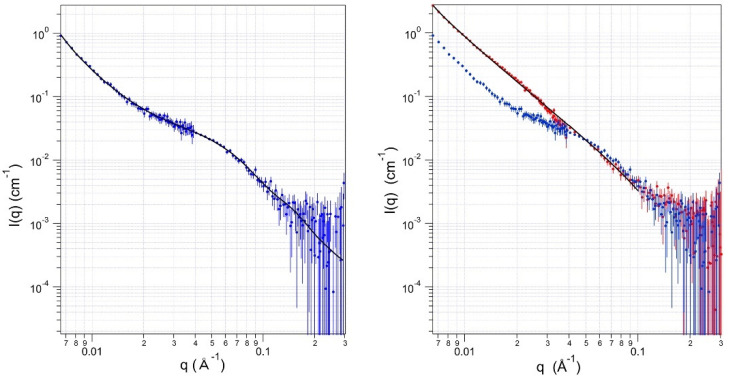
Small angle neutron scattering results for *B. anthracis* PA83 in aqueous solution at pD 7.8 (blue) or pD 4.9 (red). The lines through the data are least-squares best fits of either a right parallelepiped (pD 7.8) or a fractal power law (pD 4.9). The error bars represent the standard deviations of the neutron count mean values.

To a first approximation, peaks and shoulders in plots of the scattering intensity *I*(*q*) versus the momentum transfer *q* (e.g., Figure 2) are caused by features in the objects with sizes ≈ 2π/*q.* However, the determination of the protein’s shape requires mathematical modeling. Here, we used simple geometrical sizes and shapes (spheres, cylinders, parallelepipeds) to approximate the proteins’ structures for these calculations. Shapes are assumed to be homogeneous in SLD throughout, and the total SLD is the sum of the different atomic components. In addition, the surrounding water is homogeneous at the wavelength scale but with a different, known SLD.

### 2.1. Solution Structures of PA83

**Figure 3:**
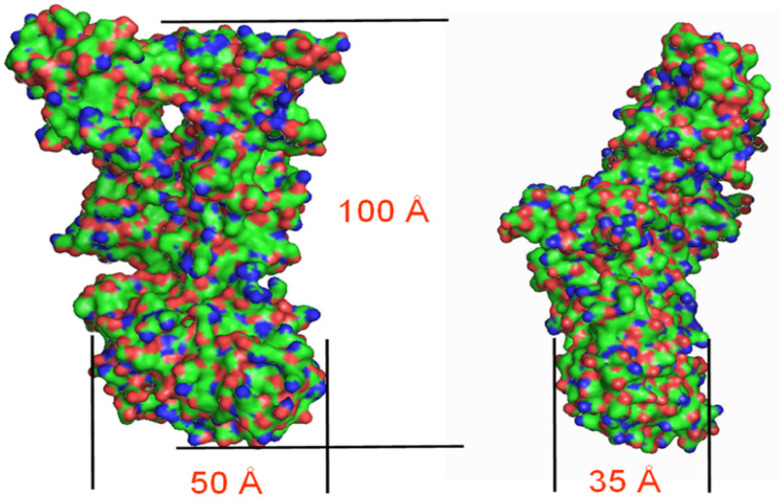
Perpendicular projections of the crystal structure of monomeric PA83 from the PDB (3TEY) [56].

Figure 2 illustrates the SANS data for PA83 in solutions at two different pD values. As seen in the main plot, at pD 7.8 (blue), the scattering data between 0.045 Å^−1^ ≲ *q* ≤ 0.3 Å^−1^ reports on structural features in the range of ≈ 20 Å to 140 Å. Table 1 includes the best-fit depth, width, and length of a homogeneous right parallelepiped to these data (black line). These dimensions are similar to the measured sizes of monomeric PA83 from its crystal structure [56] (Figure 3). The volume of a parallelepiped estimated from the SANS data is 18 Å × 63 Å × 71 Å = 80 × 10^3^ Å^3^, which is comparable, within one standard deviation, to a volume = 98.5 × 10^3^ Å^3^ calculated from the protein molecular mass (83 kg mol^−1^) and an assumed protein density (1.4 g cm^−3^) [57]. This volume estimate indicates that at pD 7.8, the PA83 scattering unit is a monomer. The scattering data for *q* ≲ 0.1 Å^−1^ are well fit by a fractal power law [58], which indicates that the solution also contains aggregates of the protein.

In contrast to the data obtained at pD 7.8, at pD 4.9 (red), the scattering data are virtually featureless and are best fit to a straight line (power-law slope = 2.25) on the log-log plot. Therefore, this suggests that PA83 forms an aggregate that is larger than the longest length of the data window, 900 Å [59]. The power-law slope fits the pD 4.9 sample data for 0.007 ≤ *q* ≤ 0.1, and the greater *q* value is equivalent to a length of 63 Å, essentially the size of a PA83 monomer. The scattering at higher *q*-values arises from the molecular structure of the PA83. However, the noise level and number of points in that range prevent us from discerning a definitive, detailed structure. The minor differences in the power-law slopes at the two pD values could be due to an artifact of the curve-fitting. Both values are commonly found for random clustering of macromolecules [58]. 

### 2.2. Solution Structure of PA63

In vivo, protective antigen is secreted as the 83 kg mol^−1^ monomeric B-toxin, PA83. However, because PA63 allows the entry of the A-toxins LF and EF into the cytoplasm, where they interfere with two cellular pathways and cause cell death [4], PA63 itself is used as a model system for anthrax toxin mechanistic studies [5,19,20,60,61,62,63]. To understand the process of how PA63 can form channels, and to test existing structural models for it, we used SANS to study PA63 in solution.

**Figure 4:**
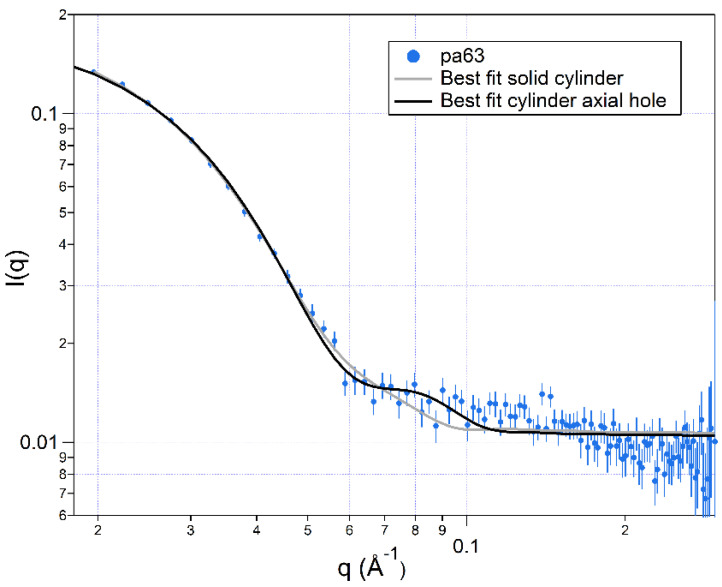
Small angle neutron scattering results for *B. anthracis* PA63 in aqueous solution. The calculated best fitting scattering curves are for a solid right cylinder (gray) or a right cylinder with an axial hole (black). The error bars represent the standard deviations of the neutron count mean values.

**Figure 5:**
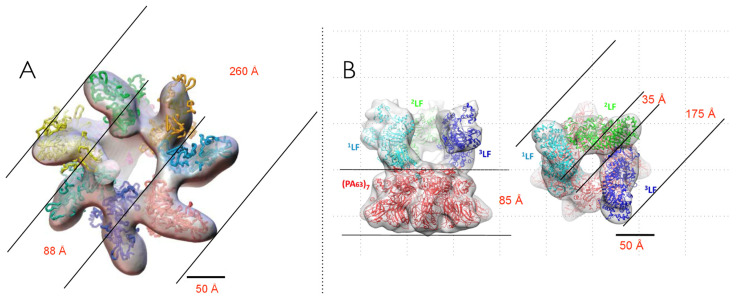
Two different structures of heptameric PA63 complexed to three LF proteins estimated from cryo-electron microscopy. The indicated dimensions are compared with the SANS results in Table 2. Images adapted from (**A**) Ren et al. [27] and (**B**) Fabre et al. [28].

Figure 4 shows the SANS results for PA63 at pD 7.2 for 0.02 Å^−1^ ≲ *q* ≲ 0.25 Å^−1^ (i.e., length scales between ≈ 310 Å and 25 Å). Note the presence of features in the scattering data at *q* ≈ 0.08 Å^−1^ (length scale ≈ 80 Å). The solid lines are the best fits to the data assuming either a solid cylinder (gray) or a hollow cylinder (black). The dimensions for the best-fit structures shown in Table 2 suggest that the structure is that of a heptameric complex, which is consistent with the claim that PA63 forms a stable heptamer in solution [64]. From the table, both the inner hole radius and the outer radius, found with SANS, are considerably different (≈2-fold smaller) than those obtained with cryo-EM by Ren and colleagues [27] (Figure 5A). In contrast, the SANS results for those dimensions are similar to those obtained with cryo-EM by Fabre et al. [28] (Figure 5B), but the heptamer’s length obtained here with SANS is ≈ 25 % less than was observed with cryo-EM [28]. 

### 2.3. PA63 Channel Formation Kinetics Measurements

We showed earlier that α-HL formed channels in planar lipid bilayer membranes at nearly constant rates over long time periods at both pH 7.5 and pH 4.5, and that the rate was ≈ 15-fold greater at the lower pH value [31]. The former result allowed us to reconstitute α-HL into tethered bilayer membranes at a sufficiently high surface concentration for neutron reflectometry structural studies of fully-functional channels [31]. Below, we describe our attempt to reproduce this process with PA63.

#### 2.3.1. Channel Formation by *B. anthracis* PA63

**Figure 6:**
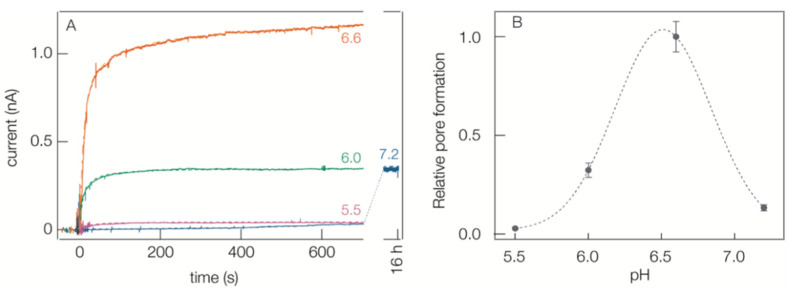
pH dependence of pore formation by *B. anthracis* protective antigen 63 (PA63). (**A**) Ionic transmembrane current time series following an injection of PA63 at *t* = 0 into the aqueous phase bathing one side of the lipid bilayer membranes. The aqueous solutions were at either pH 7.2 (blue), 6.6 (orange), 6.0 (green), or 5.5 (purple), and the applied potential was +10 mV DC. (**B**) pH dependence of pore formation estimated from the current value at 600 s (the dashed line is drawn to guide the eye). The error bars represent the standard deviations of 4 to 7 replicates of experiments on new membranes with fresh PA63 added to the chamber.

PA63 in buffered aqueous solution forms channels spontaneously when the protein is added to one of the two aqueous solutions bathing either side the planar lipid bilayer membrane [12,13,17,19,20,65]. However, at pH 8.5, no conducting channels form (data not shown). At pH 7.2 and with a constant DC applied potential, the ionic current time series in Figure 6A shows that after protein injection (*t* = 0), the current rises slowly over 16 h. In contrast, at pH 6.6, the current increases relatively rapidly in the first 30 s and more slowly thereafter. At pH 6.0, the initial current formation rate is similar to that at pH 6.6, but the current at 600 s is 3-fold less. A plot of the current levels at 600 s for different pH values, relative to that at pH 6.6, is shown in Figure 6B. The peak at ≈ pH 6.6 suggests there are at least two pH-dependent processes (one increasing and one decreasing with pH) that control PA63 channel formation. Electrochemical impedance spectroscopy was used to confirm that the decrease in the channel insertion rate also occurs in a tethered bilayer membrane on a solid support prepared as described previously in our α-HL structural study [31] (data not shown).

#### 2.3.2. AC Current Measurement

**Figure 7:**
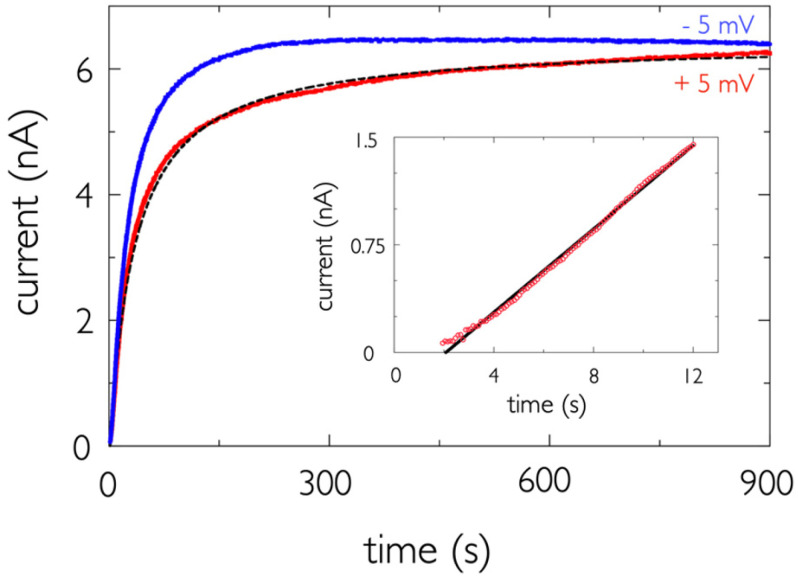
The absolute values for the resistive component of the ionic current estimated with peak applied voltages *V_applied_* = +5 mV (red) or −5 mV (blue) (10 mV peak-to-peak sine wave input potential) at pH 5.5 and an initial concentration of PA63 of 10 nM. A least-squares fit to a model that assumes the decrease in channel formation rate is caused by PA63_7_ dimerization in solution (see text below) to the +5 mV data is also shown (dashed black curve). (Inset): A plot of the initial ionic current time series for *V* = +5 mV with a linear regression fit to the data (black line).

The most striking feature in the ionic current (Figure 6A) is the near cessation of channel formation within ≈ 1 min after adding PA63 to the bilayer chamber. However, for times greater than 1 min, the current does not quite reach a steady state, which we thought might be due, in part, to an electrode polarization artifact. We, therefore, repeated the electrophysiology experiments with a 10 Hz sinusoidal applied potential and obtained essentially the same results (Figure 7). The difference in the positive and negative voltage components is likely due to voltage-dependent channel-formation rates, the non-linear I-V relationship of the PA63 channel [12], and/or a small electrode offset.

#### 2.3.3. PA63 Channel Formation Kinetics

To describe the ion current kinetics, we next consider simple models for channel formation by PA63. PA63 increases the planar lipid bilayer conductance by forming nanometer-scale pores or channels in the membrane [12,13,17,19,20,65]. If we assume that each channel in the membrane acts independently of the others, the total current is simply:

(1) $$I\left(t\right)=i_{sc}N\left(t\right)$$

where *N*(*t*) is the number of channels in the membrane at time *t*, and *i_sc_* is the current per (single) channel at a given applied potential. Given that the channels form within seconds of protein addition, diffusion of the protein from the bulk is not rate-limiting (which is discussed below). Thus, the rate of channel formation is assumed to be proportional to the bulk concentration of the channel forming unit, *C*(*t*),

(2) $$\frac{dN\left(t\right)}{dt}=k_{m}C\left(t\right)$$

where *k_m_* is an apparent rate constant for channel insertion into the membrane (number of channels M^−1^ s^−1^). If we assume there is no significant loss of protein due to aggregation, binding to the electrophysiology chamber walls, etc., then *C*(*t*) is a constant (*C*_0_), and the ionic current would be:

(3) $$I\left(t\right)=i_{sc}k_{m}C_{0}t$$

For simplicity, we assume that the channel forming unit is a PA63 heptamer [64] (Equation (4)), that channels form when PA63 heptamers in solution bind to the membrane (Equation (5)),

(4) $$7\;PA63_{solution}\;\overset{k_{h}}{\to }\;PA63_{7,solution},$$

(5) $$PA63_{7,solution}+membrane\;\overset{k_{m}}{\to }PA63_{7,membrane},$$

and these reactions proceed irreversibly. *PA*63_7,*solution*_ and *PA*63_7,*membrane*_ denote PA63 heptamers in solution or associated with the membrane, respectively. The SANS data for PA63 (Figure 4) shows that some of the PA63 in solution has dimensions consistent with a heptamer. We further assume that the heptamers form instantaneously when PA63 is added to the aqueous solution, which has a lesser pH value than that of the stock solution.

Equation (3) is consistent with α-HL channel experimental data [31] and also with the initial channel formation results reported here with PA63 (Figure 7, inset). The least-squares linear fit to this data shows that the slope is ≈0.145 nA s^−1^. Because the single-channel current is *i_sc_* ≈ 0.4 pA under these conditions [19], and we assume the initial heptamer concentration as 1/7 of the initial monomer concentration (i.e., [PA63_7_](0) = 10 nM/7 = 1.42 nM), then *k_m_* = 2.7 × 10^11^ channels M^−1^ s^−1^. In addition, channels are initially inserted into the membrane at a rate provided by the fitted slope divided by the single-channel current: 0.145 nA s^−1^/0.4 pA channel^−1^ ≈ 362 channels s^−1^.

If the reactions in Equations (4) and (5) were the only processes to occur, because the number of functional channels in the membrane is much greater than the number of proteins in solution, the protein concentration in solution should remain unchanged, and the current should increase linearly with time (Equation (3)). Because that is clearly not the case (Figure 6A and Figure 7 main plot), additional reaction schemes, which account for a decrease in either the channel formation rate or the number of functional channels (or both), are needed to describe the channel formation kinetics.

The channel formation rate, and thus the rate of change of ionic current, would decrease if the concentration of PA63 molecules in the bulk solution decreased. That could be caused by aggregation of PA63 heptamers in solution (as dimers and/or higher-order structures, as is indicated by part of the SANS data in Figure 4) or due to PA63 heptamers binding to and blocking existing PA63 channels. These irreversible schemes are described by Equations (6) and (7), respectively.

(6) $$2\;PA63_{7,solution}\;\overset{k_{D}}{\to }{\left(PA63_{7}\right)}_{2,\;solution}$$

(7) $$PA63_{7,solution}+{\left\{PA63\right\}}_{7,channel}\overset{k_{I}}{\to }{\left\{PA63\right\}}_{\mathrm{inactivated}\;\mathrm{channel}}$$

where *k_D_* and *k_I_* are forward rate constants. The reaction in Equation (7) is ruled out by inspection because if it were the sole cause of the time-dependent decrease of the channel formation rate, that would require a near-perfect balance between the channel formation and channel blocking rates to occur only after the first minute of channel formation, which is unlikely.

For simplicity, we further assume that the decrease of the PA63_7_ concentration in solution is caused solely by the formation of heptameric dimers (Equation (6)), although additional oligomerization or clustering of the protein may occur, as seen here with SANS, but we ignore that possibility here. Thus, the rate of change of the PA63_7_ concentration due to dimer formation in the bulk, *C*(*t*), can be written as:

(8) $$\frac{dC\left(t\right)}{dt}=-k_{D}C{\left(t\right)}^{2}$$

The solution to this differential equation is:

(9) $$C\left(t\right)=\frac{C_{0}}{1+k_{D}\;C_{0}\;t}$$

where *C*_0_ is the initial bulk concentration of PA63_7_.

Substituting Equation (9) into Equation (3) results in the ionic current time dependence:

(10) $$I\left(t\right)=\frac{i_{sc\;}k_{m}\;C_{0}\;t}{1+k_{D}\;C_{0}\;t}$$

As t → ∞, a predicted complete loss of PA63_7_ in solution would cause the current to asymptote towards a steady-state value $I\left(\infty \right)=i_{sc\;}k_{m}/k_{D}$, which closely resembles the observed trends (Figure 6A and Figure 7 main plot).

A least-squares fit of Equation (10) to the current (with +5 mV applied potential) is shown in Figure 7 (dashed black line), and the goodness of fit parameter is *r^2^* = 0.995. The value of *k_m_* = 3.5 × 10^11^ channels M^−1^ s^−1^ determined from the non-linear fit over the entire range of data (≈ 900 s) is surprisingly close to the value determined from the linear fit to the initial current rise (only ≈ 1.7-fold greater than *k_m_* ≈ 2.7 × 10^11^ channels M^−1^ s^−1^ obtained from the linear fit to the initial current recording, *see above*), which is remarkable. We therefore, conclude that the simple dimerization model is reasonable. In addition, the fit of Equation (10) to the data shows that *k_D_* = 2.2 × 10^7^ M^−1^ s^−1^. We are currently studying the experimental dependence of *k_m_* and *k_D_* with the initial PA63 concentration.

From Equations (2) and (9), the channel formation rate is:

(11) $$dN\left(t\right)/dt=\frac{k_{m}\;C_{0}\;\left(1-k_{D}\;C_{0}t/(1+k_{D}\;C_{0}\;t\right))}{1+k_{D}\;C_{0}\;t}$$

Using the non-linear fit parameters for *k_m_* and *k_D_*, at *t* = 5 s, the channel formation rate is 371 channels s^−1^, which is in good agreement with the estimate obtained from the linear fit to the early time ion current data above (362 channels s^−1^), suggesting that the initial-rate calculation was robust.

Because the solved expression for the channel current (Equation (10)) looks similar to a non-time dependent Langmuir adsorption isotherm [66], one might think that the channel formation rate decreases asymptotically within 10 min because the number of channel binding sites left are vanishingly small. However, that can be ruled out because, at the level of nearly saturated current (Figure 7), the channels only occupy ≈ 10^−3^ % of the membrane surface. It is also conceivable that the channel formation process nearly ceases because of competitive binding between channel-forming PA63_7_ and inactive protein).

**Figure 8:**
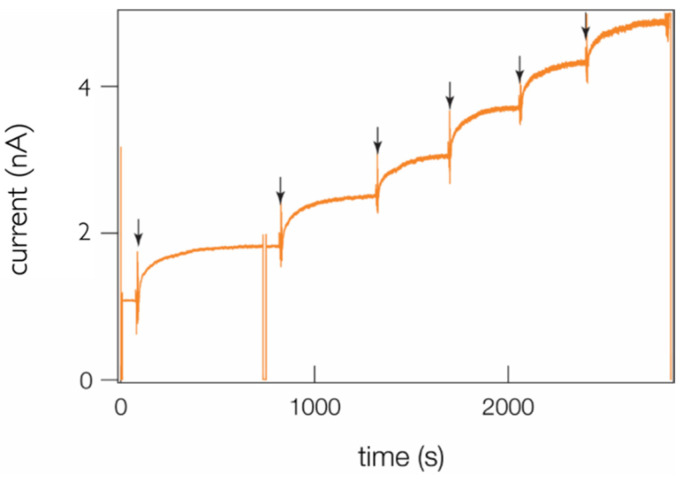
Sequential additions of fresh PA63 (arrows) cause new channel formation. Aliquots of 1 nM stock PA63 solution were added to one of the two aqueous phases bathing the membrane. The transient current drop at *t* ≈ 750 s was caused by a brief change to the applied potential (to check for electrode polarization). Variations in the current responses were caused by slight differences in the stirring duration and the time between sequential additions of fresh PA63 stock solution to the bilayer chamber.

To test both hypotheses, we made sequential injections of fresh PA63 to the same bilayer chamber compartment in a single membrane experiment (Figure 8). The data show that the addition of a new protein causes new channel formation with the same qualitative kinetics trend and magnitude increase in the current, which clearly demonstrates that the channel formation cessation is *not* caused by the loss of channel binding sites in the membrane.

## 3. Discussion

The solution structures of the two *B. anthracis* protective antigen proteins, full-length PA83, and its proteolytically cleaved product PA63, were determined with small angle neutron scattering. At pD 4.9, most of the PA83 scattering data are described by a linear slope described by a fractal power law, which indicates that most of the protein formed aggregates over a wide length scale. At pD 7.8, PA83 is a monomer and was modeled as a right parallelepiped with a volume ≈ 124 × 10^3^ Å^3^, similar to the volume estimated from its molecular mass and a typical protein density value. Some residual aggregate remains.

At pD 7.2, the PA63 SANS data were fit by a right circular cylinder with an axial through-hole. The structure is inconsistent with a cryo-EM structure of PA63_7_ bound to three LF molecules by Ren and colleagues [27] but similar to the structure of PA63_7_, also bound to three LF molecules, obtained by Fabre et al. [28]. 

With the goal of studying the interaction of the PA63 channel with *B. anthracis* lethal factor and edema factor with neutron reflectometry, as had been done for another ion channel [31], we determined the ability of PA63 to form channels in planar lipid membranes. Like its structural analog, the *S. aureus* α-HL toxin, which spontaneously forms channels in membranes at nearly complete surface coverage, PA63 also initially forms channels, but unlike α-HL, another process occurs with a time constant of ≈1 min that inhibits subsequent channel insertion. This latter phenomenon will have to be significantly reduced or eliminated to use NR for structural studies of the PA63_7_ channel itself, of the two A toxins (LF or EF) bound to the channel, and of small molecules that inhibit the binding of LF and EF to the channel.

The electrophysiology experiments were performed by vigorously stirring the solution for several seconds when the protein was added, and we assumed the protein was well dispersed by that process. The short delay (several seconds) after protein addition to the chamber suggested that complications to the ionic current kinetics due to diffusion of channel-forming units through the unstirred layer of solution adjacent to the membrane could be ignored at this time [67,68,69,70,71]. 

**Figure 9:**
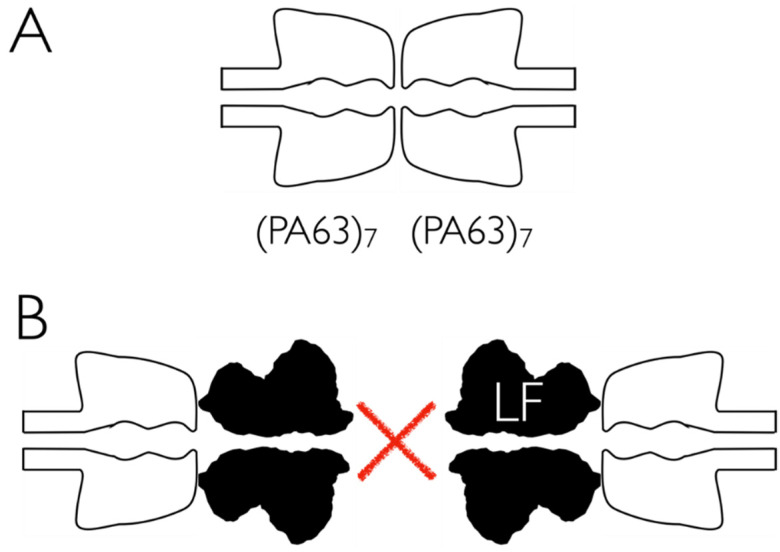
Cartoon of a possible method to inhibit the dimerization of PA63 heptamers in solution. (**A**) With PA63 heptamers only, the protein is assumed to oligomerize (dimerization shown here). (**B**) If, instead, PA63 is added to an aqueous solution that contains an excess of *B. anthracis* LF or EF, the binding of either to the channel cap domain may interfere with the formation of dimers, or perhaps even larger oligomers. The transmembrane β-barrel segment is depicted here in a non-extended configuration.

In the chemical kinetics scheme above (Equations (6), (8) and (10)), we suggested that the loss of channel-forming protein is caused initially by dimerization of PA63_7_ in solution. Higher-order oligomerization in solution is currently beyond the ability of these electrophysiology experiments to discern. Nevertheless, if dimers are formed via contacts between neighboring heptamer cap domains, as is illustrated by the cartoon in Figure 9A, it is conceivable that the addition of an excess of *B. anthracis* LF or EF (or perhaps small molecules that act as therapeutic agents against anthrax toxin infections [12]) could inhibit PA63_7_ dimerization (Figure 9B) and thereby allow for a greater concentration of PA63 channels per unit area in the membrane as desired for neutron reflectometry experiments. We are exploring this possibility.

We are also investigating (a) the stoichiometry of channel-forming units (using fluorescence-based single-molecule experiments), (b) the concentration dependence of the initial channel-formation kinetics, and (c) whether PA63_7_ in solution decreases because it binds to the excess lipid in the chamber, an artifact that is possible given the membrane formation method used here [72]. 

## 4. Conclusions

Our results show that at near-neutral pH, PA83 is a monomer in solution (it does not oligomerize as is assumed to occur on membrane-bound receptors). However, the SANS data also show that the cleaved form of PA83, PA63, forms oligomers in solution that have the same dimensions as heptamers (as judged by cryo-EM), which suggests that heptamer formation by PA63 does not critically depend on being bound to a lipid membrane. In addition, the results of the ion channel kinetic experiments demonstrate that reactions in solution prevent all the heptamers from inserting into the membrane. As a result, a different experimental protocol is needed to keep PA63 in a form that can insert unimpeded for intervals in excess of minutes.

## 5. Materials and Methods

### 5.1. Small Angle Neutron Scattering (SANS) of PA83 and PA63 Proteins in Solution

Recombinant *B. anthracis* protective antigen 63 (PA63, List Biological Laboratories, Campbell, CA, USA) was added to 99.9 % D_2_O (Cambridge Isotope Labs, Tewksbury, MA, USA) to make a solution of 1.0 mg mL^−1^ in the protein. The final solution additionally had 10 mM bisTris propane, pD 8.5, and 1.25 % trehalose. Recombinant *B. anthracis* protective antigen 83 (PA83, List Biological Laboratories) was dissolved in 95 % D_2_O to 1 mg mL^−1^, which, with the material’s 5 mM HEPES buffer, made the solution pD 7.8. Acetate buffer was used to make the pD 4.9 solution. The PA83 solutions also contained 50 mM NaCl from the original lyophilized material.

### 5.2. SANS Data Collection

The 1 mg mL^−1^ solution was held in a (1.00 ± 0.01) mm path-length cylindrical silica spectrometry cell (Hellma, Plainview, NY, USA) with a volume ≈ 320 μL. SANS measurements were performed on the NG3 and NG7 30 m SANS instruments at the NIST Center for Neutron Research (NCNR) in Gaithersburg, MD, USA [73]. Data was collected for the protein solutions and the D_2_O buffer at ambient temperature. SANS was measured using cold neutrons with wavelength λ = 6.0 Å, and Δλ/λ = 0.11.

Scattered neutrons were detected with a 64 cm × 64 cm two-dimensional position-sensitive detector with (128 × 128) pixels with 0.5 cm pixels. Raw counts for each pixel were put on the same relative scale by normalizing to an incident beam monitor count made by a detector in parallel with the data collection. The scattering was then corrected for a non-uniform detector response. Data were placed on an absolute scale by normalizing the scattering intensity to the measured incident beam flux. The data were then radially averaged to produce the scattering curves of log_10_ *I(q)* versus log_10_ *q*, where *I(q)* is the scattering intensity, *q* = (4πλ)sin θ is the momentum transfer, λ is the neutron wavelength, and 2θ is the scattering angle measured from the axis of the incoming neutron beam. Two camera positions were used for PA83: 2 m and 10 m. The sample-to-detector position at 2.0 m had a 20 cm beam stop offset, which, with the 6.0 Å wavelength, provides a *q* range 0.006 Å < *q* < 0.3 Å^−1^, equivalent to a length range of ≈900 Å to ≈21 Å.

The PA83 and PA63 scattering data were corrected for the D_2_O background by subtracting the scattering from the D_2_O buffer alone. No solute-volume correction was needed for the 1 mg mL^−1^ because any adjustment would be less than 0.1%, which is within the uncertainties of the scattering amplitudes. PA83 scattering data were collected at pD 7.8 and 4.9. Under the conditions used here, the measured pH and pD are the same [74]. 

### 5.3. SANS Curve Fitting

The scattering curves were fit using Igor Pro software with fitting macros developed at the NCNR [75]. For PA63, two geometric models were compared: either a homogeneous cylinder (two geometric variables) or a homogeneous cylinder with an axial hole (three geometric variables). All scattering curve fittings account for the neutron optics by a smearing algorithm and assigning the flat, incoherent background left after subtracting the buffer scattering. For PA83, the geometry of solitary molecules was modeled as a right parallelepiped, and, where appropriate, a fractal slope model was applied.

### 5.4. Electrophysiology: Membrane Formation and Channel Activity

Electrolyte solutions were prepared with 0.1 M KCl, 10 mM of a buffering agent, and either 2-(*N*-morpholino)ethanesulfonic acid, 3-(*N*-morpholino)propanesulfonic acid, sodium phosphate, or sodium dihydrogen phosphate in 18.2 MΩ cm water (UHQ reagent-grade purification system Millipore, Billerica, MA, USA). The solution pH was adjusted with either citric acid or hydrochloric acid (Sigma-Aldrich, St. Louis, MO, USA).

The “solvent-free” phospholipid bilayer membranes [72] were formed with 1,2-diphytanoyl-sn-glycero-3-phosphocholine (DiPhyPC; Avanti Polar Lipids, Inc., Birmingham, AL, USA), in high purity pentane (Burdick and Jackson, Muskegon, MI, USA) on a ≈100 μm diameter hole in a 17 μm thick Teflon partition that separated two 2.5 mL Teflon wells [44]. A transmembrane potential was applied via two matched Ag/AgCl electrodes (In Vivo Metric, Healdsburg, CA, USA) separated from the solution by Vycor glass frits (Koslow Scientific Company, Englewood, NJ, USA). The current was converted to voltage and amplified with an Axon Instruments 200B patch-clamp amplifier (Molecular Devices, Sunnyvale, CA, USA) in the voltage-clamp mode and then digitized with an Axon 1440 16-bit analog to digital converter. The PA63 was injected from a 1 mg mL^−1^ stock solution to the indicated concentrations (Figure 6, Figure 7 and Figure 8) with the solution vigorously stirred for several seconds with Teflon-coated magnetic flea bars. A FORTRAN program was written to remove the header, minimize the stirring artifacts, and check for drift in the conductance at the end of each run. The data was typically saved as 2-byte integers (binary), in some cases median filtered and subsequently scaled to the correct ionic current values. Some of the current time series data were fitted to chemical kinetic models using TableCurve 2D (Systat, San Jose, CA, USA). To increase the dynamic range of the current recordings, the data in Figure 7 were initially acquired with a high-gain setting, and when the current was close to saturating the patch-clamp amplifier, the gain was lowered to the next setting. This was repeated several times over the course of each experiment.

**Figure 10:**
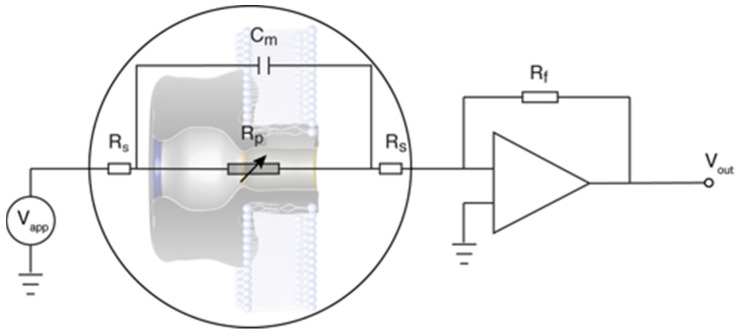
Illustration and electrical circuit analog of the electrophysiology experiments. For DC applied potentials, the membrane capacitance (C_m_) and the Teflon membrane support capacitance (not shown) in parallel with C_m_ have no effect on the DC current. However, for time-varying applied potentials, the total capacitance is sufficiently large for the capacitive reactance to contribute appreciably to the time-varying current. The membrane current is converted to a voltage (V_out_) via a FET amplifier with a feedback resistor (R_f_). The DC series resistance, R_s_, due to the electrodes and electrolyte solution is much less than the resistance of the PA63 channels in the membrane (i.e., R_s_ ≪ R_p_).

Figure 10 shows a cartoon illustration of the electrophysiology setup. In most cases, the applied potential was direct current, and positive potentials were defined in reference to the virtual ground on the side opposite to which the protein was added (i.e., positive potentials drive cations into the cap domain side of the channel). To test for the effects of voltage-induced channel inactivation and electrode polarization, we also employed a 10 Hz sine wave alternating voltage across the membrane. For those experiments, an additional program was written to extract the resistive component of the current.

## Figures and Tables

**Table 1:** Geometrical parameters for fitting PA83 scattering at 1 mg mL^−1^ protein in D_2_O ^a^.

Sample/Model	Depth (Å)	Width (Å)	Length (Å)	Power Law Slope
pD 7.8/Right parallelepiped	18 ± 4	63 ± 3	71 ± 4	2.64 ± 0.04
pD 4.9/Power function	- - ^b^	- -	- -	2.255 ± 0.0004

^a^ The uncertainties listed are found when the other geometric variables are held constant. ^b^ In the power law fit, the protein’s Depth, Width, and Length are not determined.

**Table 2:** Geometrical parameters for fitting PA63 scattering at 1 mg mL^−1^ protein in D_2_O.

Model	Hole Radius (Å)	Outside Radius (Å)	Length (Å)
Homogeneous cylinder	- - ^a^	74 ±1	63 ± 3
Homogeneous cylinder w/axial cylindrical hole	19 ± 2	67 ± 1	71 ± 3
Figure 5A, cryo-EM, PA63_7_ complexed with 3 LF molecules [27]	44	130	- -
Figure 5B, cryo-EM, PA63_7_ complexed with 3 LF molecules [28]	19	88	85

^a^ In the homogenous cylinder model, the radius is not determined.

## Data Availability

Raw SANS data were generated at the NIST Center for Neutron Research. Data can be accessed at ftp://129.6.120.51/pub/sansdata. Reduced and corrected SANS scattering data supporting the findings of this study are available from the corresponding authors upon reasonable request.
